# Predicting high-grade prostate cancer at initial biopsy: clinical performance of the ExoDx (EPI) Prostate *Intelliscore* test in three independent prospective studies

**DOI:** 10.1038/s41391-021-00456-8

**Published:** 2021-09-30

**Authors:** Erik Margolis, Gordon Brown, Alan Partin, Ballentine Carter, James McKiernan, Ronald Tutrone, Phillipp Torkler, Christian Fischer, Vasisht Tadigotla, Mikkel Noerholm, Michael J. Donovan, Johan Skog

**Affiliations:** 1 Urology Center of Englewood, Englewood, NJ USA; 2 Delaware Valley Urology, Vorhees, NJ USA; 3 Johns Hopkins Hospital, Baltimore, MD USA; 4 Columbia University Medical Center, New York, NY USA; 5 Chesapeake Urology Associate, Baltimore, MD USA; 6 Exosome Diagnostics, a Bio-techne brand, Martinsried, Germany; 7 Exosome Diagnostics, a Bio-techne brand, Waltham, MA USA; 8 University of Miami, Miami, Florida USA

**Keywords:** Cancer, Biomarkers

## Abstract

**Background:**

The ability to discriminate indolent from clinically significant prostate cancer (PC) at the initial biopsy remains a challenge. The ExoDx Prostate (*IntelliScore*) (EPI) test is a noninvasive liquid biopsy that quantifies three RNA targets in urine exosomes. The EPI test stratifies patients for risk of high-grade prostate cancer (HGPC; ≥ Grade Group 2 [GG] PC) in men ≥ 50 years with equivocal prostate-specific antigen (PSA) (2–10 ng/mL). Here, we present a pooled meta-analysis from three independent prospective-validation studies in men presenting for initial biopsy decision.

**Methods:**

Pooled data from two prospective multi-site validation studies and the control arm of a clinical utility study were analyzed. Performance was evaluated using the area under the receiver-operating characteristic curve (AUC), negative predictive value (NPV), positive predictive value (PPV), sensitivity, and specificity for discriminating ≥ GG2 from GG1 and benign pathology.

**Results:**

The combined cohort (*n* = 1212) of initial-biopsy subjects had a median age of 63 years and median PSA of 5.2 ng/mL. The EPI AUC (0.70) was superior to PSA (0.56), Prostate Cancer Prevention Trial Risk Calculator (PCPT-RC) (0.62), and The European Randomized Study of Screening for Prostate Cancer (ERSPC) (0.59), (all *p*-values <0.001) for discriminating GG2 from GG1 and benign histology. The validated cutoff of 15.6 would avoid 23% of all prostate biopsies and 30% of “unnecessary” (benign or Gleason 6/GG1) biopsies, with an NPV of 90%.

**Conclusions:**

EPI is a noninvasive, easy-to-use, urine exosome–RNA assay that has been validated across 3 independent prospective multicenter clinical trials with 1212 subjects. The test can discriminate high-grade (≥GG2) from low-grade (GG1) cancer and benign disease. EPI effectively guides the biopsy-decision process independent of PSA and other standard-of-care factors.

## Introduction

Prostate cancer (PC) is the most common cancer in men and the second cause of cancer-related death in the United States. In 2021, it is estimated that approximately 249,000 men will be diagnosed with PC and 34,000 will die of their disease [1]. As mentioned in the National Comprehensive Cancer Network (NCCN) 2020 Prostate Cancer Early Detection guidelines, nearly 20 million men in the United States will engage in PC early detection discussions due to anxiety associated with fluctuating PSA levels, a positive family history, and race.

Importantly, since PSA is not a reliable biomarker for the identification of clinically significant Grade Group 2 (GG2) and higher disease, there will continue to be a large percentage of men with either benign biopsies or clinically indolent, low-volume GG1 PC in addition to men with a more advanced stage. This cycle will result in a high fraction of men experiencing procedures such as surgery, radiation, or additional biopsies as part of an active-surveillance program [1–6]. Furthermore, although there have been improvements in the prostate biopsy process—including the use of prophylactic antibiotics—the procedure is not entirely benign and rare complications may result, including an infection rate of 3–5% with the potential for emergency-room visits and hospitalizations [7].

The ExoDx (EPI) Prostate *Intelliscore* test (Exosome Diagnostics, Waltham, MA) is a first-catch urine exosome-based liquid-biopsy assay that has been independently validated in two prospective multi-site studies and the standard-of-care control arm of a utility study that evaluated EPI in the biopsy decision process [8–10]. Utilizing the expression levels from three genes (i.e., PCA3, ERG, and SPDEF), EPI provides a risk score predicting whether a patient presenting for their first biopsy with an equivocal PSA from 2 to 10 ng/mL is likely to have GG2 or greater (high-grade) prostate cancer. The EPI risk score is independent of all clinical variables and performed without the need for a digital rectal exam or prostate massage [11]. The absence of clinical variables in the EPI algorithm represents an important differentiator from other assays predicting high-grade prostate cancer, including 4 K (OPKO Diagnostics, Miami, FL) and SelectMDX (MDx Health, Irvine, CA). Furthermore, EPI relies on the exosome RNA signature instead of clinical factors already part of the biopsy-consideration process [12–14]. The EPI signature as a test to discriminate risk of high-grade PC (HGPC) on initial prostate biopsy was first evaluated on a cohort of 453 subjects in 2015 [11], followed by a multicenter prospective validation study in 2016 [8], which included 519 men from 22 community practices and academic urology sites. In this study, a cutoff score of 15.6 avoided 27% of biopsies with an NPV of 91% for detection of Gleason score (GS) 7 and higher. The majority of missed subjects (9 out of 12) were low-volume (<3 cores) GS 3 + 4 and 3 subjects with a dominant Gleason pattern 4. In addition, an alternative recommended cutoff score of <20 avoided 40% of unnecessary biopsies and 31% of total biopsies, with a NPV of 89%. A second validation study and the control arm of a utility study representing 503 and 190 subjects, respectively, confirmed the original results [9, 10]. All three studies had similar clinical performance and outperformed standard of care.

The 2020 National Comprehensive Cancer Network (NCCN) Prostate Cancer Early Detection V2.2020 guidelines and the updated United States Preventive Services Task Force (USPSTF) [15] both acknowledge the benefits of informed and patient-specific (i.e., age, life expectancy, and patient history) PSA screening as a mechanism to reduce overdiagnosis and overtreatment of indolent prostate cancer. This has resulted in the need for additional noninvasive tests that not only reduce the number of unnecessary biopsies but also maintain a sufficient sensitivity to identify clinically significant disease. Pooled results from three independent, multisite, prospective-validation studies in the intended-use population confirmed performance of the EPI test in identifying clinically significant prostate cancer on initial biopsy.

## Subjects and methods

### Study design and population

The studies included subjects that had never been diagnosed with PC, were 50 years or older with PSA 2–10 ng/mL, and scheduled for their first prostate-needle biopsy. The three study protocols were previously approved by the respective local institutional review boards and all subjects had provided informed consent. At each of the sites, the developed protocol and associated statistical analysis plan(s) were agreed upon by individual principal investigators prior to patient consent and trial-data collection. Subjects were enrolled sequentially and received site-specific standard of care. In approximately 90% of subjects, an MRI was not utilized, and the subjects received a 12-core transrectal ultrasound (TRUS) biopsy. Of note, only 9.3% of the subjects in the second validation study [9] and no subjects from the utility study had a concurrent MRI as part of the biopsy-decision process. By report, the primary rationale for the lack of an MRI was cost and accessibility. A correlation of EPI results with biopsy pathology using both the 15.6 and alternative 20 EPI-test result cutoff points was performed.

### Assay methods

First-catch urine samples (15–20 mL) were collected and stored at 4 °C for up to 14 days (majority were received within five days) prior to shipping to a central laboratory (Exosome Diagnostics, Inc., Waltham, MA). All sites received a urine-collection container and shipping kits; men from the first validation study had received a pediatric urine cup with instructions to only collect urine within the requested volume range, while a standardized 20-mL volume-restricted vessel was used in the second validation cohort and the utility study. Methods used in exosome isolation, extraction of RNA, and reverse transcriptase polymerase chain reaction (RT-PCR) were previously published [8–11]. The test provides a binary low- or high-risk score based on a validated cutoff point of 15.6 (scale 0–100) that predicts the presence of GG2 or higher prostate cancer [8]. The 15.6 cutoff was also externally confirmed by five urologists and a machine-learning biostatistician using training and prevalidation clinical cohorts with an emphasis on the percentage of missed GG2 and GG3. Since the level of acceptable risk for missing HGPC vs. benefits of reducing biopsies (i.e., sensitivity vs. specificity) is different among urologists, an alternative cutoff point of 20 was also assessed. We had previously demonstrated that men with a score ≤15.6 (or ≤20) are less likely to have GG2 or higher prostate cancer on a subsequent biopsy.

#### Statistical methods

The primary objective of this pooled analysis was to evaluate combined performance of the EPI test for predicting GG2 or higher PC on a first biopsy for men with a PSA 2–10 ng/mL in a merged cohort that consisted of three prospective, multisite trials. We also employed the PSA measurements alone as well as the Prostate Cancer Prevention Trial Risk Calculator 2.0 (PCPT-RC 2.0) and the European Randomized Study of Screening for Prostate Cancer (ERSPC) RC to further establish and compare the diagnostic risk of prostate cancer [16]. Receiver-operating characteristics for all models assessed clinical performance. Subgroup analyses, including restricted age of 55–69 years as per United States Preventive Services Task Force (USPSTF) [15] and the 3 ng/mL PSA cutoff as per NCCN 2.2020 guidelines, were also evaluated for EPI performance.

A pooled meta-analysis was conducted on the merged cohort. Area under the curve (AUC) of the ROC was assessed for EPI, PCPT-RC, ERSPC, and PSA with TRUS biopsy outcome being used for subject labeling. Sensitivity, specificity, negative predictive value (NPV), and positive predictive value (PPV) are reported for the EPI cutoff points of 15.6 and 20. Where applicable, confidence intervals were calculated using the Clopper–Pearson method. DeLong’s test was applied to assess the significance of AUC differences between analyses. We also evaluated the net health benefit of the EPI test for predicting GG2 PC across a range of clinical preferences, which represents how physicians value different outcomes for their subjects. The datasets analyzed during the current study may be available from the corresponding author upon reasonable request.

## Results

### Study population

There were 1212 eligible study subjects enrolled from three prospective trials: Validation 1, *n* = 519 [8], Validation 2, *n* = 503 [9], and Validation 3 [10], *n* = 190 enrolled across 23 community-practice sites and four academic medical centers (i.e., Columbia University Medical Center, New York, New York; Johns Hopkins University, Baltimore, MD; University of Michigan, Detroit, MI; and NYU Langone, New York, NY [complete listing of sites in Supplemental Table S1]). The median age was 63 years with a median PSA of 5.2 ng/mL; 18% of subjects self-reported a positive family history of PC and 70% identified as Caucasian and 17% African American. The digital rectal exam (DRE) was nonsuspicious in 87% and suspicious in 13%. See Table 1 for complete demographic and clinical characteristics of individual and pooled validation subjects.

**Table 1:** Pooled cohort demographic and clinical characteristics.

	Pooled cohort	1st Validation (JAMA oncology 2016)	2nd Validation (European urology 2018)	3rd Validation/utility study (PCAN 2020)
Total patient N	1212	519	503	190^a^
Age median, IQR	63 (58, 69)	64 (58, 68)	64 (59, 69)	63 (58, 69)
PSA median, IQR	5.2 (4.3, 6.6)	5.1 (4.3, 6.4)	5.4 (4.4, 6.7)	5.2 (4.4, 6.7)
Family History				
Yes	223 (18.4%)	117 (22.5%)	72 (14.3%)	34 (17.9%)
No	987 (81.4%)	402 (77.5%)	431 (85.7%)	154 (81.1%)
NA	2 (0.2%)	0 (0%)	0 (0%)	2 (1%)
Ethnicity				
African American	204 (16.8%)	87 (16.8%)	71 (14.1%)	46 (24.2%)
Asian/Pacific Islander	37 (3.1%)	13 (2.5%)	18 (3.6%)	6 (3.2%)
Caucasian	854 (70.5%)	377 (72.6%)	350 (69.6%)	127 (66.8%)
Other	96 (7.9%)	35 (6.8%)	52 (10.4%)	9 (4.8%)
NA	21 1.7(%)	7 (1.3%)	12 (2.4%)	2 (1.1%)
DRE				
Nonsuspicious	888 (73%)	352 (67.8%)	379 (75.3%)	157 (82.6%)
Suspicious	155 (13%)	77 (14.8%)	63 (12.5%)	15 (7.9%)
NA	169 (14%)	90 (17.3%)	61 (12.1%)	18 (9.5%)
Grade Group				
Benign	585 (48.3%)	268 (51.6%)	234 (46.5%)	83 (43.7%)
GG 1 (GS3 + 3)	261 (21.5%)	103 (19.8%)	111 (22.1%)	47 (24.7%)
GG 2 (GS3 + 4)	198 (16.3%)	84 (16.2%)	86 (17.1%)	28 (14.7%)
GG 3 (GS4 + 3)	84 (6.9%)	36 (6.9%)	26 (5.2%)	22 (11.6%)
GG 4 (GS8)	39 (3.2%)	11 (2.1%)	26 (5.2%)	2 (1.1%)
GG 5 (> GS8)	45 (3.7%)	17 (3.3%)	20 (4.0%)	8 (4.2%)

^a^Patients from the control arm with biopsy outcome results.

### Biopsy Gleason grading and grade group, GG classification

The median number of TRUS-guided biopsy cores was 12 for all 1212 subjects with biopsy diagnoses performed at individual site-designated pathology practices. There was no central pathology review included in the three validation studies. The total positive biopsy rate was 52%: 21.5% GG1, 30% ≥GG2, and 13.8% ≥GG3 (see Table 1). Although PSA and age were comparable across all studies, there was a 8% increase in the positive biopsy rate from the first to third validation study (48–56%), a 3% increase in the diagnosis of ≥GG2 PC (28–31%), and a 12–17% increase in clinically significant ≥GG3 disease. These mild increases may reflect the 2012 USPSTF recommendation against PSA screening [17].

#### EPI as a predictor of≥GG2 prostate cancer in the merged cohort

**Fig. 1:**
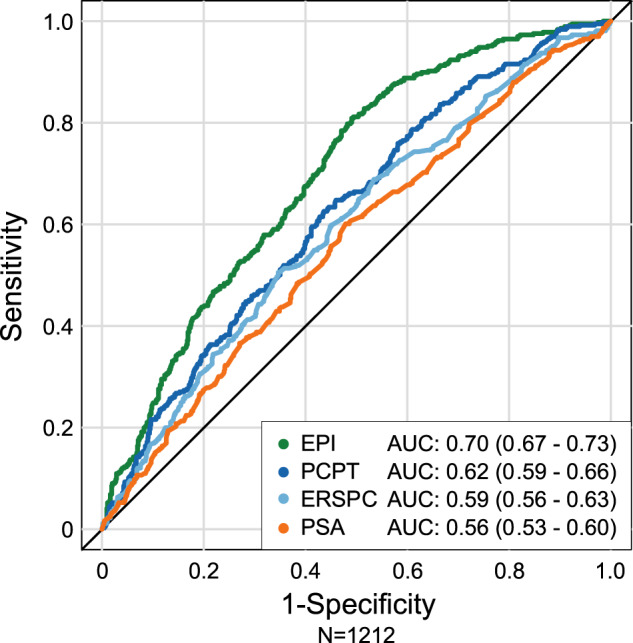
Pooled EPI performance using the area under the receiver operator characteristic (AUC) curve. Area under receiver operating characteristic (AUC) curves illustrates performance of pooled EPI (*n* = 1212) to discriminate HGPC vs. PCPT-RC, ERSPC RC, and PSA.

The EPI test in the pooled cohort exhibited comparable performance as in the previous individual validation studies. The pooled cohort EPI AUC of 0.70 (95% CI 0.67–0.73) was superior to the PCPT-RC 2.0 AUC 0.62 (95% CI 0.59–0.66), ERSPC-RC AUC 0.59 (95% CI 0.56–0.63), and PSA AUC 0.56 (95% CI 0.53–0.60) (Fig. 1). The DeLong test comparing the differences between AUC curves further demonstrated good independent performance of the EPI test (Table 2).

**Table 2:** Performance comparison (AUC, 95%CI) of the EPI test to alternative models and DeLong test for significance. EPI has a significantly higher AUC than all alternative models.

Test/Model	*N* = 1022	
	AUC (95%CI)	*p*-value
EPI	0.70 (0.67–0.73)	–
PCPT2.0-RC	0.62 (0.59–0.66)	<0.001
ERSPC	0.59 (0.56–0.63)	<0.001
PSA	0.56 (0.53–0.60)	<0.001

The pooled EPI-validated cutoff point of 15.6 would avoid 23% of all biopsies and 30% of the true-negative biopsies with an NPV of 90%, PPV 36.4%, and a sensitivity of 92% (Table 3). The alternate cutoff point of 20 increased the avoided biopsies to 34% with an NPV of 89%, PPV of 40%, and a sensitivity of 87% (Table 4). Across the total cohort (*n* = 1212), only 2.3% (28/1212) or 3.8% (46/1212) of subjects would experience delayed detection (≥GG2) at the <15.6 or <20 threshold, respectively.

**Table 3:** Performance of the EPI test with a cut point of 15.6 in the pooled cohort.

	EPI ≥ cut point	EPI < cut point	Total	Performance	(95%CI)
Biopsy positive/≥GG2	338	28	366	Sensitivity, 92.3%	(89.1–94.9)
Biopsy negative/GG1	591	255	846	Specificity, 30.1%	(27.1–33.4)
Total	929	283	1212	PPV, 36.4%	(33.3–39.6)
				NPV, 90.1%	(86.0–93.3)
Prevalence	30.2%	Predicted negative	23.3%

**Table 4:** Performance of the EPI test with a cut point of 20 in the pooled cohort.

	EPI ≥ cut point	EPI < cut point	Total	Performance, %	(95%CI)
Biopsy positive/≥GG2	320	46	366	Sensitivity, 87.4	(83.6–90.6)
Biopsy negative/GG1	486	360	846	Specificity, 42.6	(39.2–46.0)
Total	806	406	1212	PPV, 39.7	(36.3–43.2)
				NPV, 88.7	(85.2–91.6)
Prevalence	30.2%	Predicted negative	33.5%		

Performance of the 15.6 or 20 cutoff point with respect to GG3 or higher disease was also evaluated. An EPI score of ≥15.6 identified 93% (156/168) of GG3 cancers. If the EPI score was <15.6, the chance of missing a ≥GG3 on biopsy was 4% (i.e., missed 12 of 283). Alternatively, a cutoff point of ≥20 identified 88% (149/168) of GG3 with the chance of missing 19 subjects or 11% false negatives. If the EPI score was <20, the chance of missing a ≥GG3 cancer was 5% (19/406). The 12 or 19 subjects missed by the cutoff points of 15.6 and 20 represent 3% and 5% of the overall ≥GG2 population (12/366; 19/366, respectively) and 1% and 1.5% of the entire cohort of 1212 men. These data further support the use of the 15.6 cutoff point for the majority of men currently being evaluated for a prostate biopsy. Performance for GG2 vs. GG3 at the 15.6 vs. 20 cutoff point is shown in Supplemental Table S2.

Similar results were observed for predicting either a ≥GG2 or ≥GG3 biopsy when applying the USPSTF age restrictions of 55–69 years. The percent false-negative rate for ≥GG3 with the 15.6 cutoff point was <5% in both the USPSTF and NCCN-recommended subgroups (Supplemental Figs. S1, S2 and Tables S3, S4).

**Fig. 2:**
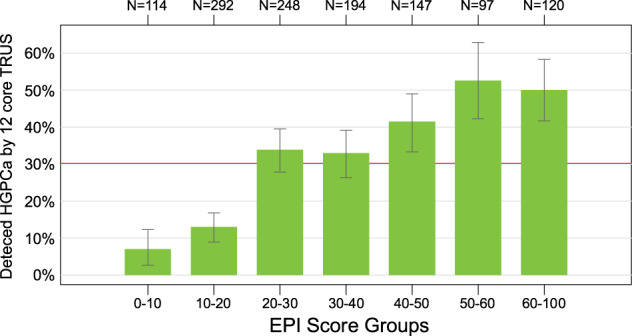
EPI score and probability of HGPC on a TRUS prostate biopsy. Bar-chart illustration of binned EPI scores and the probability of finding HGPC on a subsequent TRUS 12-core biopsy.

**Fig. 3:**
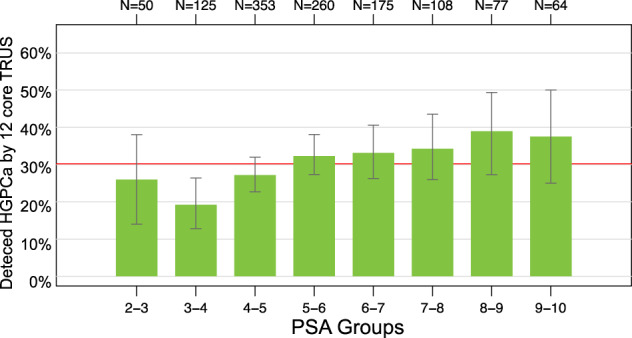
PSA values and probability of HGPC on a TRUS prostate biopsy. Bar-chart illustration of binned PSA values and the probability of finding HGPC on a subsequent TRUS 12-core biopsy.

The probability of identifying ≥ GG2 PC based on the EPI score was determined in the pooled cohort. As shown in Fig. 2 and Supplemental S3, EPI score and risk (or probability) of finding HGPC had a linear relationship with an EPI score from 20 to 60, where the probability of finding HGPC is EPI over 50% at an EPI score >50. It is important to note that the likelihood of finding GG2 or higher PC is limited by the 12-core TRUS biopsy, which has a sensitivity of approximately 48–50% for finding HGPC, therefore, the probability of finding cancer beyond 50% will be limited [18]. Furthermore, stratifying PSA groups did not further discriminate HGPC risk in the 2–10 ng/mL range (Fig. 3).

**Fig. 4:**
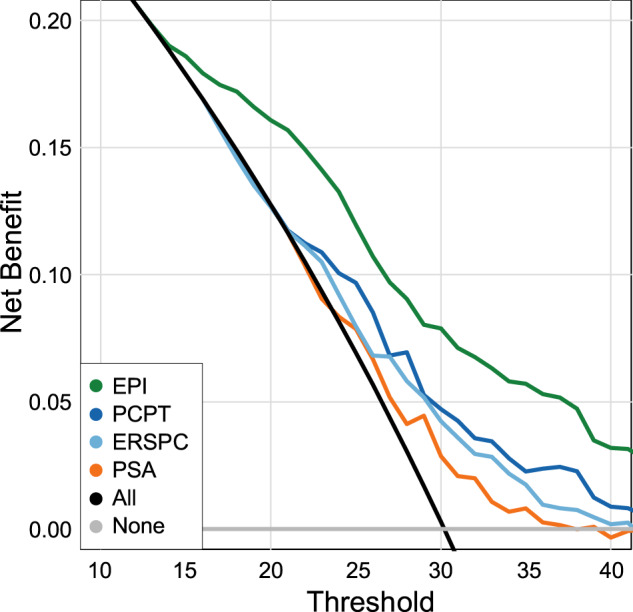
Pooled EPI Decision-curve analysis. Decision-curve analysis demonstrating net clinical benefit of EPI in the overall biopsy-decision process.

We also investigated the net clinical value of the EPI test using a decision-curve analysis by comparing EPI scores with PCPT-RC V2.0, ERSPC-RC, and PSA variables over a range of probabilities (Fig. 4). The net benefit is the sum of true positives minus false negatives in reference to the biopsy-decision threshold. In this analysis, the EPI test had the highest net benefit across the 10–40% biopsy-decision threshold, demonstrating a significant clinical utility when compared with more traditional methods.

In summary, the EPI test maintained performance for predicting GG2 or higher prostate cancer on initial biopsy for a large cohort of men (*n* = 1212) across three independent prospective studies. Higher EPI scores were associated with a greater probability of GG2 on a subsequent biopsy with an overall net clinical benefit when compared with a standard-risk calculator and PSA. The risk of missing GG3 was stable at 3% in the pooled analysis with the 15.6 cutoff point. Taken together, the EPI score gives a personalized risk assessment, stratifying subjects into lower or higher risk for HGPC in a population where PSA and other clinical factors are nondiscriminatory.

## Discussion

The prostate biopsy-decision process is multifactorial and encompasses an integration of clinical variables, including a PSA history, DRE exam, family history, race, and patient/urologist anxiety. PSA has merits as a screening biomarker with a positive impact on PC mortality; however, the downside has been an increase in the overall number of prostate biopsies performed in combination with the overdiagnosis of low-grade, (presumably) non-clinically significant disease. With approximately two million transrectal ultrasonography-guided prostate biopsies (TRUS-Bx) performed each year in the United States and Europe [19], there is increasing concern over the risk of multi-drug-resistant infections, which was recently reported to be approximately 10% [20]. Collectively, there is much to be gained by introducing more objective, biomarker-driven assays into the biopsy-decision paradigm. While clinical assessment tools, such as the PCPT-RC, have value in assessing risk, these clinical calculators are not designed to be patient specific.

A screening strategy that preferentially targets GG2 PC and higher, with an additional emphasis on ≥GG3 disease while avoiding detection of GG1 and benign pathology, has the potential to maintain the mortality reduction while reducing harm from overdetection of indolent PC. We have previously confirmed clinical performance of a noninvasive, urine-based gene expression assay, EPI, to discriminate ≥GG2 cancer from GG1 and benign disease for men aged ≥50 years undergoing initial biopsy with PSA levels 2–10 ng/mL [8–10]. In the current analysis, we confirmed and extended the previous studies with pooled data from three independent validation studies (designated V1, V2, and V3) representing 1212 subjects. We found that the EPI test, a gene signature within exosomes analyzed from voided urine, was consistently predictive of GG2 PC with AUCs greater than PSA and the PCPT-RC V2.0 (AUC 0.70 > 0.56 and 0.62, respectively). In addition, the validated cutoff point of 15.6, achieved an NPV of 90%, PPV of 36%, sensitivity of 92%, and specificity of 30%. The NPV and sensitivity performance of the pooled analysis was comparable to the previous independent analyses (cutoff point 15.6; V1, NPV 91%, sensitivity 92%, V2, NPV 89%, sensitivity 93%, and V3, NPV 87%, sensitivity 92%) [8–10].

The urine-exosome signature is derived from genes known to play a role in prostate cancer initiation and progression, including ERG, PCA3, and SPDEF [11]. To address recent developments in PSA screening, we further evaluated the 59–65-year age restriction as proposed by the USPSTF and the PSA cutoff of 3 ng/mL, as proposed by NCCN, and found comparable performance. The 2018 USPSTF-adjusted recommendation was designed to foster a more personalized, patient-specific approach to PSA screening [15]. A test that is able to reduce the “diagnosis” of low-grade and/or low-risk disease on a patient-specific basis should have a positive effect on individual urologist practice-pattern variability.

Commercially available assays in the NCCN 2020 guidelines, specifically in the initial-biopsy setting, including Prostate Health Index (PHI) [21] (Beckman Coulter), SelectMDx (MDxHealth) [14], and the 4 K Score (OPKO, Inc.) [13], have varying accuracy to predict HGPC. Collectively, the assays are limited by composition of their respective validation cohorts, specificity issues of the kallikrein family, especially in the PSA 2–10 ng/mL range (e.g., PHI, 4 K), relative importance of clinical features in test (algorithm) performance, requirement of a DRE prior to collection, and additional specimen processing (e.g., Progensa, SelectMDx). Furthermore, there are limited data to support reliable discrimination of GG2 vs. GG3 on initial biopsy. Additional challenges include the integration into busy clinical practices and whether the respective assays have been recommended for both the initial- and repeat-biopsy setting. Of note, neither of the well-established online clinical risk calculators (i.e., PCPT-RC, v2.0, and ERSPC-RC) were able to effectively discriminate HGPC in the current meta-analysis.

In the pooled cohort, applying the 15.6 cutoff point would avoid 23% of all biopsies (i.e., <15.6 cutoff point) and 30% (i.e., specificity) of the true-negative biopsies with an NPV of 90%, PPV 36.4%, and a sensitivity of 92%. The alternate cutoff point of 20 increased biopsy avoidance to 34% with an NPV 89%, PPV 40%, and a sensitivity of 87%. Of clinical importance is that across the total cohort (*n* = 1212), only 2.3% or 3.8% of subjects would experience delayed detection of ≥GG2 at the <15.6 or <20 threshold, respectively, and for GG3, only 1–1.5% would potentially have a delayed detection at either cutoff point. These results are comparable to prior validation studies for missing both ≥GG2 and ≥GG3 disease. The ability to subclassify GS7 into both GG2 and GG3 categories supports the clinical distinction of a dominant pattern 4, which impacts directly on patient outcome and management plan [22]. It is widely accepted that not all GG2 cancers behave similarly and the volume of the dominant pattern 4 dictates disease course. The implication is that subjects with a low-volume (<10%) pattern-4 cancer are most likely to behave as a dominant pattern 3 and are appropriate for active-surveillance protocols [23]. Additional studies are underway to further refine this exosome signature, including the introduction of additional clinical variables such as race and tumor genetics.

EPI provided an overall net benefit (i.e., predicting ≥ GG2) when compared with standard clinical tools. This was further elucidated in the decision-curve analysis, where EPI—when compared with important clinical variables such as PSA level and the PCPT-RC and ERSPC risk calculators—demonstrated a higher net benefit beginning at a biopsy-decision threshold probability of 10%, which was maintained up to a maximum of 40%. As EPI performance is based on gene expression only, there is an option for the urologist to introduce other parameters, such as obesity, underlying genetics, and race for developing a more personalized risk assignment.

The pooled data also illustrate that as the EPI score increases above 20, the risk or probability of GG2 prostate cancer increases in a linear fashion up to an EPI score of 60, where the probability of finding HGPC is just over 50%. It is challenging to have a linear improvement of a biomarker beyond 50%, since TRUS biopsy is known to miss roughly 50% of HGPC [18].

A limitation of the validation studies was the absence of a central pathology review; however, the intent was to assess real-world experience of the assay with site-directed pathologists. Another missing component was the lack of a multiparametric MRI as part of the overall assessment process. Most sites across the three studies did not routinely use MRI in the initial biopsy setting during this period. Future studies will incorporate EPI in determining the use of MRI and inclusion of the EPI-risk score into an algorithm that includes the PI-RADS designation and other clinical variables.

## Conclusion

In summary, the EPI test performed equally well across three independent prospective studies involving 23 community practices and 4 academic medical centers across the United States. The EPI test was superior to PSA, PCPT-RC, and ERSPC for predicting clinically significant GG2 and GG3 PC on initial biopsy. The results further support that increasing EPI score above 20 linearly increases the probability for finding HGPC upon an initial TRUS biopsy, up to a 50% risk at EPI scores >50.

## Supplementary information

Supplemental Materials

## Data Availability

The datasets during and/or analyzed during all reported studies may be available from the corresponding author upon request.
