# Supplementary material for: Predicting high-grade prostate cancer at initial biopsy: clinical performance of the ExoDx (EPI) Prostate Intelliscore test in three independent prospective studies
Source: Prostate Cancer Prostatic Dis. 2021 Sep 30;25(2):296–301. doi: 10.1038/s41391-021-00456-8 (PMC9184274; doi:10.1038/s41391-021-00456-8)

**Supplementary Material**

**TABLE S1. List of Study Sites**

| **Site** | **Number Enrolled** | **Percent of total** |
| --- | --- | --- |
| 21st Century Oncology | 16 | 1.32 |
| Associated Medical Professionals of N.Y. PLCC | 9 | 0.74 |
| Associated Urologists of North Carolina | 114 | 9.41 |
| AUC | 23 | 1.9 |
| Carolina Urologic Research Center | 2 | 0.17 |
| Chesapeake Urology Bel Air | 16 | 1.32 |
| Chesapeake Urology Brandywine Medical Center | 15 | 1.24 |
| Chesapeake Urology Columbia | 8 | 0.66 |
| Chesapeake Urology Columbia Midtown Medical Bldg | 8 | 0.66 |
| Chesapeake Urology Franklin Square 1 | 12 | 0.99 |
| Chesapeake Urology Franklin Square 2 | 1 | 0.08 |
| Chesapeake Urology GBMC | 14 | 1.16 |
| Chesapeake Urology Good Samaritan Hospital | 6 | 0.5 |
| Chesapeake Urology Hanover | 7 | 0.58 |
| Chesapeake Urology Havre de Grace Medical Center | 4 | 0.33 |
| Chesapeake Urology Silver Springs | 4 | 0.33 |
| Chesapeake Urology St Joesphs Medical Center | 3 | 0.25 |
| Chesapeake Urology St. Agnes Hospital | 7 | 0.58 |
| Chesapeake Urology Towson Bellona Ave | 23 | 1.9 |
| Chesapeake Urology Union Memorial Hospital | 8 | 0.66 |
| Chesapeake Urology Westminster | 7 | 0.58 |
| Chesapeake Urology Woodholme Medical Center | 25 | 2.06 |
| Columbia University Medical Center | 2 | 0.17 |
| Comprehensive Medical Center | 90 | 7.43 |
| Delaware Valley Urology | 132 | 10.89 |
| Erlanger Institute for Clinical Research | 16 | 1.32 |
| Five Valleys Urology | 57 | 4.7 |
| Johns Hopkins University | 104 | 8.58 |
| Manhattan Medical Research | 5 | 0.41 |
| Metropolican Urologic Services | 1 | 0.08 |
| NYU Urology Associates | 25 | 2.06 |
| Premier Medical Group of the Hudson Valley | 10 | 0.83 |
| Premier Urology Group | 26 | 2.15 |
| Radiation Oncology of San Antonio | 1 | 0.08 |
| Regional Urology - Cross Creek/Greenville Health System | 21 | 1.73 |
| Southeastern Research Group | 1 | 0.08 |
| Southeastern Urology Associates | 1 | 0.08 |
| University of Michigan Health System | 7 | 0.58 |
| University Urology Associates | 8 | 0.66 |
| Urologic Consultants of Southeast Pennslyvania | 51 | 4.21 |
| Urologic Research and Consulting LLC | 137 | 11.3 |
| Urological Consultants division of Chesapeake Urology | 3 | 0.25 |
| Urological Consultants division of Chesapeake Urology Bethesda | 10 | 0.83 |
| Urological Consultants division of Chesapeake Urology Germantown | 1 | 0.08 |
| Urological Consultants division of Chesapeake Urology Wheaton | 8 | 0.66 |
| Urology Center Research Institute | 47 | 3.88 |
| Urology Clinics of North Texas | 31 | 2.56 |
| Urology Health Specialists | 10 | 0.83 |
| Urology of Virginia | 31 | 2.56 |
| Urology of Virginia, PLLC | 41 | 3.38 |
| UTHSCSA - Dept. of Urology | 3 | 0.25 |
|  |  |  |
| **Total** | **1212** | **100.04** |

**Figure S1**. Area under receiver operating characteristic (AUC) curves illustrate performance of pooled EPI in the USPSTF age restricted cohort, 55-69 years, (n=833) vs. PCPT-RC, ERSPC RC and PSA to discriminate HGPC.

**Figure S2**. Area under receiver operating characteristic (AUC) curves illustrate performance of pooled EPI in the NCCN PSA 3ng/mL and age 45-75 year restricted cohort (n=1097) vs. PCPT-RC, ERSPC RC and PSA to discriminate HGPC.

**Table S2**: Performance of the EPI test to rule out ≥GG2 and ≥GG3 HGPC at the 15.6 vs 20 cut-point.

|  | Detection of HGPC (≥GG2) | | Detection of HGPC (≥GG3) | |
| --- | --- | --- | --- | --- |
|  | **EPI 15.6** | **EPI 20** | **EPI 15.6** | **EPI 20** |
| Cohort size N | 1212 | 1212 | 1212.0 | 1212.0 |
| Prevalence | 30.2 | 30.2 | 13.9 | 13.9 |
| % Samples < Cutpoint | 23.3 | 33.5 | 23.3 | 33.5 |
| Sensitivity | 92.3 | 87.4 | 92.9 | 88.7 |
| Specificity | 30.1 | 42.6 | 26.0 | 37.1 |
| NPV | 90.1 | 88.7 | 95.8 | 95.3 |
| PPV | 36.4 | 39.7 | 16.8 | 18.5 |

**Table S3**: Performance of the EPI test with a cut point of 15.6 in the pooled cohort with USPSTF constraints.

|  | **EPI** $\boldsymbol{\geq}$**cut-off point** | **EPI < cut-off point** | **Total** | **Performance** | **(95%CI)** |
| --- | --- | --- | --- | --- | --- |
| Biopsy Positive/>GG2 | 223 | 16 | 239 | Sensitivity, 93.3% | (89.4 – 96.1) |
| Biopsy Negative/GG1 | 422 | 172 | 594 | Specificity, 29.0% | (25.3 – 32.8) |
| Total | 645 | 188 | 833 | PPV, 34.6% | (30.9 – 38.4) |
|  |  |  |  | NPV, 91.5% | (86.5 – 95.1) |
| **Prevalence** | 28.7% | **Predicted negative** | 22.6% |  |  |

**Table S4**: Performance of the EPI test with a cut point of 15.6 in the pooled cohort with NCCN constraints.

|  | **EPI** $\boldsymbol{\geq}$**cut-off point** | **EPI < cut-off point** | **Total** | **Performance** | **(95%CI)** |
| --- | --- | --- | --- | --- | --- |
| Biopsy Positive/>GG2 | 298 | 28 | 326 | Sensitivity, 91.4% | (87.8 – 94.2) |
| Biopsy Negative/GG1 | 542 | 229 | 771 | Specificity, 29.7% | (26.5 – 33.1) |
| Total | 840 | 257 | 1097 | PPV, 35.5% | (32.2 – 38.8) |
|  |  |  |  | NPV, 89.1% | (84.6 – 92.6) |
| **Prevalence** | 39.7% | **Predicted negative** | 23.4% |  |  |

**Figure S3.** Linear regression analysis of EPI score to risk of HGPC on biopsy. Coefficient of determination (R2): 0.91Mean squared error: 0.00226


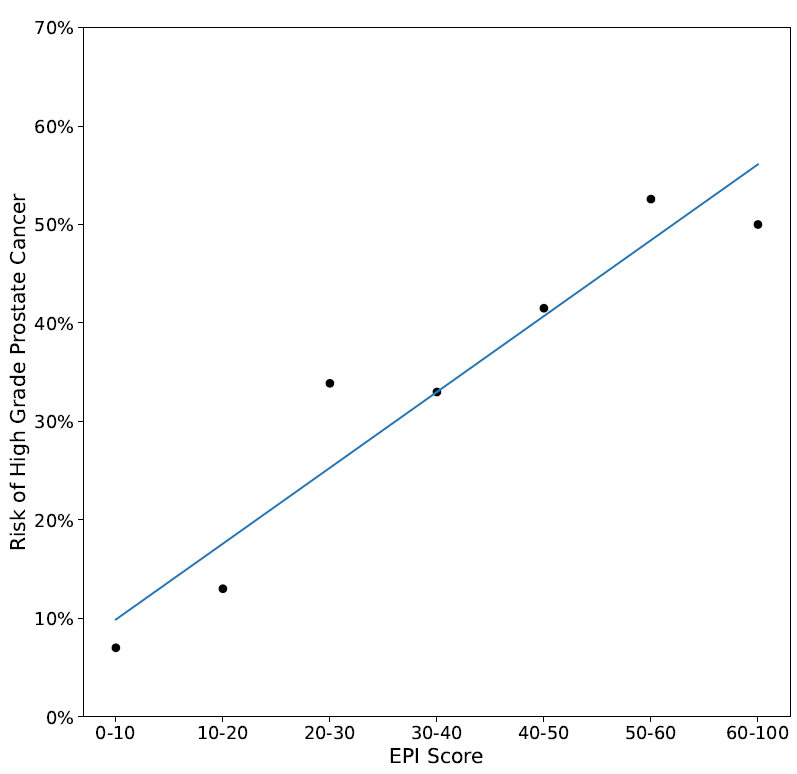

Supplement: Supplementary file 1 — Supplemental Materials [file 41391_2021_456_MOESM1_ESM.docx]
